# Knee Extensor Structure and Function in Children, Adolescents, Adults, and Older Adults With Obesity: A Systematic Review and Meta‐Analysis

**DOI:** 10.1111/obr.13949

**Published:** 2025-05-19

**Authors:** Michael N. Vakula, Youngwook Kim, Eadric Bressel

**Affiliations:** ^1^ Department of Kinesiology and Health Science Utah State University Logan Utah USA; ^2^ Department of Sports Medicine Soonchunhyang University Asan South Korea

**Keywords:** muscle, muscle function, muscle quality, muscle structure, obesity

## Abstract

**Objective:**

This study aims to evaluate the association between obesity and both absolute and relative measures of knee extensor muscle structure and contractile function across different age groups, including children, adolescents, adults, and older adults.

**Methods:**

A search for potential studies was performed in four electronic databases. Data were meta‐analyzed using a random‐effects model for our primary outcomes of knee extensor structure (muscle size and quality) and function (maximal force/torque, rapid torque, and fatigue) and compared between those with or without obesity in each age group.

**Results:**

The findings indicate that obesity significantly increases absolute measures of knee extensor maximal contractile function and muscle size. However, obesity was associated with a decrease in relative measures of maximal and rapid contractile function and muscle quality. The association of obesity with these muscle characteristics varied by age group, with the effects on knee extensor structure and function diminishing with age.

**Conclusions:**

This systematic review and meta‐analysis demonstrate that while obesity enhances absolute knee extensor muscle size and maximal force/torque, it detrimentally affects relative muscle function and quality, particularly related to activities of daily living. These effects are less pronounced in older adults, suggesting that age modulates the impact of obesity on muscle structure and function. The findings underscore the importance of interventions targeting the improvement of relative muscle function and quality in individuals with obesity. Further research is necessary to better understand these relationships and to develop more effective intervention strategies for obesity.

**Registration:**

This protocol was prospectively registered in the Open Science Framework (DOI: 10.17605/OSF.IO/ZGUK6).

## 1 Introduction

Obesity is a chronic condition characterized by excessive accumulation of adipose tissue, posing health risks across all age groups, including children, adolescents, adults, and older adults. Adult obesity is typically defined as a body mass index (BMI; kg/m^2^) of ≥ 30, while childhood and adolescent obesity is classified based on criteria such as the World Health Organization (WHO) Growth Reference or BMI‐for‐age percentiles from the Centers for Disease Control and Prevention (CDC) [1, 2, 3]. The global prevalence of obesity has surged dramatically, increasing nearly 80% from 1980 to 2015, with an estimated 107.7 million children and 603.7 million adults classified as obese in 2015 [4, 5]. Notably, obesity during childhood and adolescence often persists into adulthood, amplifying lifelong risks of cardiovascular disease, diabetes, cancer, and musculoskeletal disorders [6].

Beyond these systemic health risks, obesity significantly alters skeletal muscle structure and function, particularly in load‐bearing muscles such as the knee extensors. These muscles, essential for activities of daily living (ADLs) and postural stability, exhibit distinct adaptations in individuals with obesity. Absolute measures of muscle size and strength, such as cross‐sectional area (CSA) and peak torque, are often increased in obese individuals due to the mechanical overload associated with excess body mass [7, 8, 9]. However, relative measures, normalized to body weight or fat‐free mass, tend to be reduced, reflecting impaired muscle quality, defined as the force or power generated per unit of muscle size [10, 11, 12, 13]. For example, relative knee extensor strength is reported to be 20%–30% lower in individuals with obesity when scaled to body weight, and 6%–18% lower when scaled to fat‐free mass [8, 9, 14].

This reduction in muscle quality is attributed to intramuscular fat infiltration, which disrupts contractile properties and increases muscle stiffness [15, 16]. Additionally, obesity negatively impacts muscle contractile function, as evidenced by slower rates of torque development, greater volitional fatigue, and reduced power output during dynamic tasks [9, 11]. These impairments are not merely structural but directly influence functional performance, increasing the risk of mobility limitations and falls. For instance, intermuscular fat deposition, as measured by ultrasound echo intensity, correlates with lower internal knee extension moments during walking [11].

Despite substantial research, the literature lacks consensus on the precise association of obesity with knee extensor structure and function. While some studies report enhanced absolute muscle strength and power in obese populations, others find no significant differences, particularly when examining rapid force production (e.g., rate of torque development) or fatigue resistance [10, 11, 12, 14]. Variability in methodological approaches, such as the choice of normalization method (e.g., body mass vs. fat‐free mass) or focus on specific muscle groups, contributes to these inconsistencies. Previous reviews have explored obesity's general effects on skeletal muscle, but none have focused exclusively on the knee extensors or used meta‐analytic methods to quantify these effects.

The knee extensors are particularly important for activities requiring controlled eccentric and concentric contractions, such as walking, standing up, and recovering from a slip. These muscles are critical during the early stance phase of the gait cycle, where they absorb forces acting on the knee joint, and during postural recovery from perturbations [17, 18]. Understanding how obesity affects their structure and function is essential for designing interventions to improve mobility and reduce fall risk in this population.

This systematic review and meta‐analysis aims to evaluate the association of obesity with both absolute and relative measures of knee extensor muscle structure (e.g., size and texture) and function (e.g., maximal torque, rate of torque development, and fatigue) in children, adolescents, adults, and older adults. By synthesizing findings from diverse age groups and standardizing outcome measures, this study seeks to address inconsistencies in the literature and provide actionable insights for clinical practice and future research.

## 2 Materials and Methods

The current systematic review and meta‐analysis was created in agreement with the Preferred Reporting Items for Systematic Reviews and Meta‐analysis (PRISMA) statement [19]. To ensure that our methods were reproducible and bias was limited in the methods we utilized in the review, this systematic review and meta‐analysis was prospectively registered in the Open Science Framework prospective register of systematic reviews (DOI: 10.17605/OSF.IO/ZGUK6).

### 2.1 Eligibility Criteria

- *Types of studies*: Original research (i.e., cross‐sectional studies and randomized control trials) that investigated at least one measure of knee extensor structure or function comparing individuals with obesity and normal weight. All publication dates were considered for inclusion, and included studies were limited to those published in peer‐reviewed journals in the English language. If a randomized control trial or intervention study were to meet the eligibility requirements, only the baseline data from the study were included in the meta‐analyses.
- *Types of participants*: Children 6–10‐years old, adolescents 11–18‐years old, adults 19–64‐years old, older adults 65‐years of age or older with or without obesity. Adults and older adults with normal weight or obesity were defined as having a BMI of 18.5–24.9 or greater than 30.0, respectively. Whereas children and adolescents with normal weight and obesity were defined by any of the criteria set by the CDC, WHO, or the IOTF [1, 2, 3, 20]. Obesity was defined using BMI as it is the most commonly reported and standardized metric in the literature, allowing for consistent classification and comparability across studies. Body fat percentage (%BF), while informative, was not used due to inconsistent/insufficient reporting and variability in measurement techniques. Participants classified as overweight were not included in the current study, as our primary aim was to examine differences between normal weight and obese groups. Additionally, many studies did not report data for overweight participants as a separate category, limiting the feasibility of including this group in the analysis. Participants with cognitive dysfunction, sensory impairments, or physical disabilities that could affect physical testing were excluded from being included in the current review.
- *Types of interventions*: Studies comparing absolute or relative measures of knee extensor structure and function between individuals with obesity and those with normal weight in children, adolescents, adults, or older adults.
- *Types of outcome measures*: Primary outcome measures include absolute and relative measures of knee extensor muscle structure (size and quality) and contractile function (peak torque/force, power/RTD, and fatigue). These outcomes specifically refer to the quadriceps femoris group, which includes the rectus femoris, vastus lateralis, vastus medialis, and vastus intermedius.

### 2.2 Search Strategy

The systematic search was conducted independently by two researchers (M.V. and Y.K.) using the following electronic databases: PubMed (MEDLINE), CINAHL (EBSCO), Scopus, and Cochrane Library. Additional manual searches of reference lists in relevant articles were performed to identify studies that may not have been captured during the electronic database search. The search covered studies published up to December 21, 2024.

To ensure a comprehensive search, the strategy primarily focused on title, abstract, and keywords fields within each database. In instances where broader coverage was deemed necessary to capture studies that may not have been indexed under these fields, a supplementary full‐text search was conducted. Boolean operators and truncation were applied to refine the search strategy, and manual searches of reference lists were performed to further minimize the risk of missing relevant articles.

The search strategy was tailored to each database to account for differences in search functionalities. The general search string used was as follows: (knee extens* OR leg extens* OR quadricep* OR lower‐extremity OR lower‐limb OR knee joint OR leg muscle) AND (muscle strength OR muscle power OR muscle weakness OR muscle deficit OR muscle performance OR muscle function OR muscle structure OR muscle quality OR muscle activation OR torque OR force OR rate of force development OR rate of torque development) AND (overweight OR obes* OR body mass index OR BMI).

### 2.3 Study Selection

Two reviewers (M.V. and Y.K.) working independently of one another performed the eligibility assessment. Disagreements that arose between reviewers were resolved with a single meeting in which the eligibility of articles was discussed until an agreement between reviewers was reached.

### 2.4 Data Extraction

Data from the included studies were extracted by two independent reviewers (M.V. and Y.K.). Extracted data were recorded onto a custom Microsoft Excel document. Any data not reported in the studies were requested to the corresponding authors.

The following data were extracted from each of the studies included in the review and meta‐analyses: (1) participant characteristics (sample size, age, sex, body mass index, and body fat %), (2) type of intervention (limb analyzed, testing device, contraction type, joint angle of testing, and data normalization), (3) absolute outcome measures (isometric peak torque, isokinetic peak torque, isotonic peak torque, isokinetic fatigue, isokinetic power, RTD, and muscle size), and (4) relative outcome measures (isometric peak torque, isokinetic peak torque, RTD, fatigue, and muscle quality). Muscle quality was broadly defined as the structural and functional efficiency of muscle tissue, and the extracted variables included strength‐to‐mass ratio (torque relative to CSA or volume), echo intensity (as a proxy for intramuscular fat), and fat infiltration measured via imaging.

### 2.5 Risk of Bias

The methodological quality and risk of bias of the studies included in the systematic review were examined using a modified epidemiological appraisal instrument (EAI) developed by Genaidy et al. [21]. The original instrument consists of 43 items across five domains: study description, subject selection, measurement quality, data analysis, and generalization of results. The modified version utilizes 30 criteria of the uppermost importance with respect to the included studies (Table S1), similar to previous systematic reviews and meta‐analyses [22].

The decision to use the modified EAI, rather than more conventional tools such as Cochrane ROB2, was based on the methodological diversity of the included studies. Cochrane ROB2 is tailored to randomized controlled trials and does not comprehensively address observational study designs, such as cross‐sectional studies, which comprised a significant portion of our dataset. The modified EAI provides a more adaptable framework for assessing both internal and external validity across a range of study types, ensuring consistency and transparency in the quality assessment process.

The appraisal instrument has demonstrated good to excellent validity and inter‐rater reliability (Kappa coefficient = 90% [CI; 87% to 92%]) [21]. Two independent assessors (M.V. and Y.K.) blinded to the author and details of the publication assessed each study. Disagreements between assessors were settled during a single consensus meeting. The Cohen kappa statistic was used to calculate the level of agreement between assessors' scores for each question of the EAI [23]. The general quality of the included studies was based on the average score across each item, with a maximum possible score of 2.0. Studies were classified by the following criteria: high quality (≥ 1.4), moderate quality (1.1 to < 1.4), or poor quality (< 1.1) [21].

### 2.6 Meta‐Analysis

The primary outcome measures were the standardized mean differences in measures of knee extensor muscle structure and function comparing children, adolescents, adults, and older adults with and without obesity.

First, the means and standard deviations of the outcome measures were extracted from the included articles. For data presented in a graphical format (i.e., muscle quality [24], peak torque [25], fatigue [26], and all included outcome measures [7, 9, 27]) rather than in a table, ImageJ software (National Institutes of Health, Bethesda, MD) was used to digitize and extract the mean and standard deviation of the outcomes. When studies reported outcome data for both limbs, at multiple testing angles, or at various testing speeds, a single study‐wise effect size was calculated using a fixed‐effects model in which the condition‐wise effect sizes were weighted by their inverse variances [28]. A fixed‐effects model was selected because a common effect of obesity was expected on outcome measures within a study. When studies reported multiple subgroups with obesity or normal weight, they were combined according to the formula outlined in the Cochrane Handbook [28]. When studies reported outcome measures relative to body weight and fat‐free mass, priority was given to the measure normalized to fat‐free mass. This prioritization is due to differences in strength being largely attributable to individual differences in body composition, and fat‐free mass is a gross indication of the amount of muscle mass an individual has [10]. When studies reported multiple measures of muscle quality (i.e., MRI, ultrasound, and force/CSA), priority was given to imaging techniques (e.g., ultrasound) over force/CSA measurements. Likewise, prioritization was given to isometric measures of contractile function when studies also reported isokinetic or isotonic measures. This prioritization was due to the contrived muscle actions experienced during isokinetic testing.

The extracted data were entered into RevMan 5.4.1 software (Cochrane) to calculate overall and age‐categorized pooled effect sizes, 95% confidence intervals, and heterogeneity statistics using a random‐effects model with a restricted maximum likelihood approach [29]. A random‐effects model, rather than a fixed‐effects model, was selected due to the expected variability in experimental factors (i.e., limb or limbs analyzed, BMI group, and knee extensor analysis instrumentation). The *I* ^2^ statistic, or the percentage of variability in the effect estimate due to heterogeneity rather than chance, was used to quantify inconsistencies across studies [28]. Data were categorized into two groups of related outcome measures: (1) absolute measures of knee extensor structure and function; maximal contractile function (i.e., peak force/torque), rapid contractile function (i.e., power and RTD), and muscle size; (2) relative measures of knee extensor function and structure; maximal contractile function (i.e., peak force/torque), rapid contractile function (i.e., power and RTD), muscle fatigue, and muscle quality.

Measures of isometric, isokinetic, or isotonic peak torque/force were grouped together for analysis, as they aim to capture the “maximal or peak‐capacities” of the knee extensors function. Similarly, measures of knee extensor power (i.e., isokinetic knee extension) and RTD (i.e., isometric knee extension) were grouped together for analysis, as they aim to capture the “rapid or rate‐based capacities” of the knee extensors contractile function. Absolute and relative outcome data are presented as separate forest plots to aid in visual examination of the data between individuals with and without obesity.

In order to examine whether the summary outcome effects vary in relation to the age category of participants, pre‐specified subgroup analyses were conducted. The risk of publication bias was assessed visually using funnel plots and statistically using Egger's test [30]. Additional sensitivity analyses were conducted to assess the robustness of the findings. This process involved quantifying the changes in outcomes by excluding a single trial with an extreme value that was notably outlier compared to the other included trials within each meta‐analysis.

The following subgroups were combined into a single group to maximize the number of participants included in the analysis and control for sampling bias using the Cochrane formula in the following studies [28]: men's and women's groups [31, 32, 33], older and younger adult groups [27], and participants with Class II and Class III obesity [34]. Additionally, a single study‐wise effect size was calculated in the following studies: early and late RTD [11], right and left limb outcome measures [16, 35], isokinetic testing speeds [9, 24, 33, 36], isometric testing angles [9], rectus femoris, vastus lateralis, and vastus medialis anatomical CSA [7, 37].

## 3 Results

### 3.1 Literature Search

**FIGURE 1:**
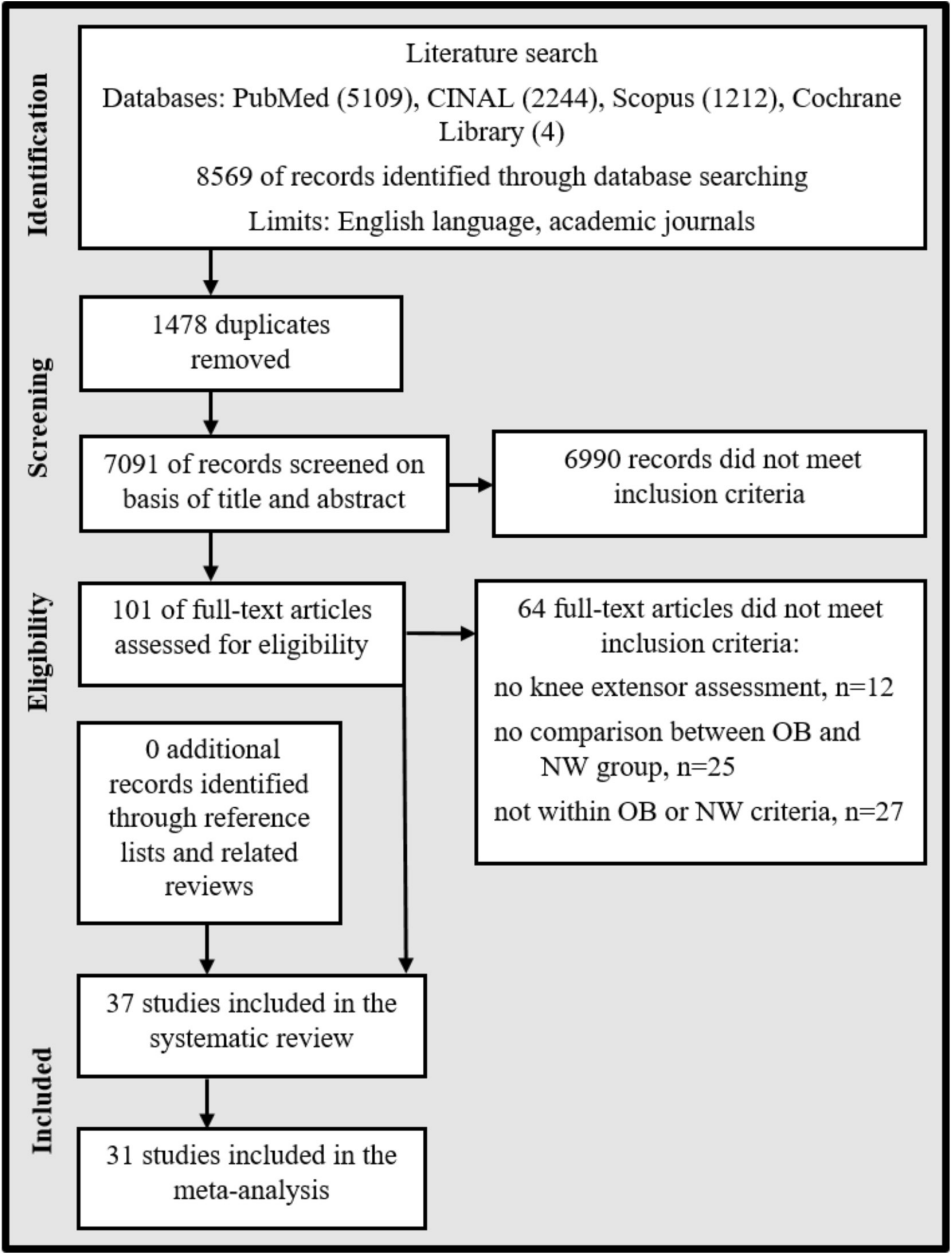
Search flow diagram of article inclusion process. NW: normal weight; OB: obesity.

The four databases that were searched resulted in 7091 distinctive articles, 37 of which met the inclusion criteria. No additional articles were identified through manual searching of related reference lists (Figure 1).

### 3.2 Assessment of Methodological Quality and Risk of Bias

The studies included in the systematic review (Table S2) had a high average quality score of 1.53 ± 0.16 out of the possible 2.0 points with a range of 1.17–1.79 points (Figure S1). The item assessing environmental covariates and confounders was not applicable to any of the included studies. Six out of the 37 included studies reported reliability data for their outcome measures. Similarly, sample size calculations were only reported in eight studies. Agreement between raters ranged from strong (i.e., 0.81) to perfect, with an average Cohen Kappa value across all studies of 0.90 ± 0.04.

### 3.3 Characteristics of Studies

There were 1413 participants with obesity and 1664 with normal weight among the 37 studies included in the systematic review (Table S3). Participants ranged in average age from eight to 80‐years old with two studies reporting on children, nine on adolescents, 17 on adults, and nine on older adults. Participants were solely females in nine studies, entirely males in seven studies, and 19 were a mix of genders. Body fat percentage ranged from 26.6% to 47.9% for individuals with obesity and from 13.6% to 33.5% for individuals classified as normal weight. Knee extensor function was measured by isokinetic dynamometry in 29 studies with the Biodex System III being the most utilized model. In all studies reporting isokinetic measures of knee extensor function, only concentric contractions were reported. Finally, at least one measure of knee extensor structure or function was normalized in 26 of the 37 studies included in the systematic review.

Relative measures of knee extensor structure and function were assessed using normalization techniques (Table S3). Relative muscle size was derived by normalizing CSA or volume to body mass or fat‐free mass. Relative muscle quality was calculated as the ratio of force (e.g., peak torque) to muscle size (CSA or volume), with ultrasound echo intensity included as a proxy for intramuscular fat infiltration. For contractile function, relative peak torque, power, and RTD were normalized to body mass or fat‐free mass, and relative fatigue was assessed as the percentage decline in torque or force during a fatiguing task.

### 3.4 Meta‐Analysis

To ensure accurate representation and minimize sampling bias, overlapping studies were carefully evaluated. Tsiros et al. [16] and Tsiros et al. [38] were identified as reporting data from identical participant groups, and thus, Tsiros et al. 2016 was excluded from the meta‐analysis. Similarly, while the NW groups in Arieta et al. [39] and Giuliani et al. [40] reported identical demographic and outcome data, indicating that they were based on the same participant cohort, the OB groups differed in both demographic and outcome measure data, suggesting they involved separate participant samples. Accordingly, the NW group was treated as a single cohort, and mean and standard deviation values from the OB groups were pooled using formulas for independent samples as recommended by the Cochrane Handbook [28]. This pooling was applied to absolute measures of knee extensor size and measures of knee extensor muscle quality.

### 3.5 Absolute Measures of Knee Extensor Structure and Function

**FIGURE 2:**
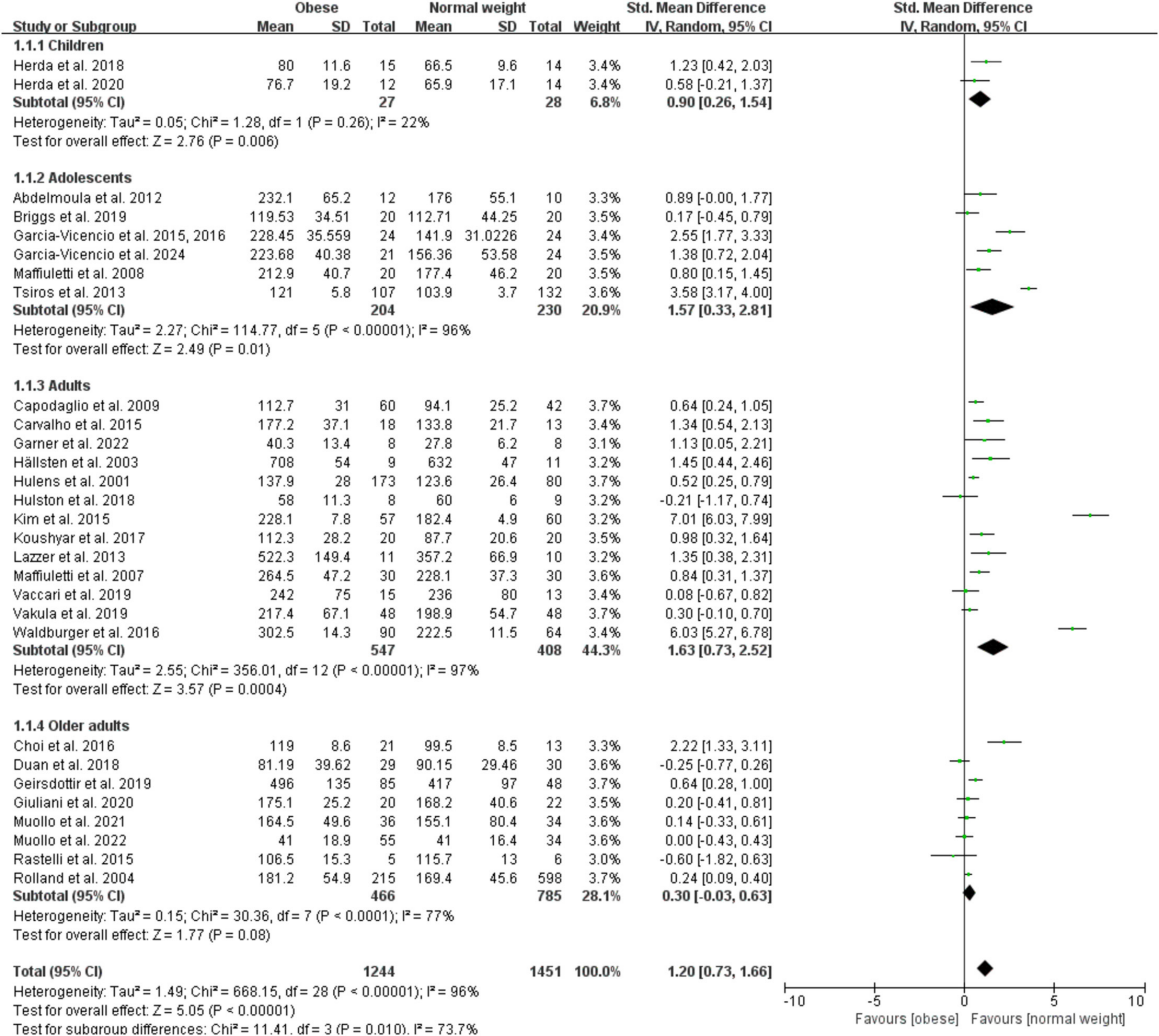
Forest plot of absolute measures of maximal knee extensor contractile function.

Obesity significantly increased absolute maximal measures of knee extensor contractile function with a pooled effect size of 1.20 (95% CI: 0.73, 1.66; *p* < 0.001; I^2^ = 96%) for the 29 studies included in the analysis (Figure 2). The funnel plot was symmetrical, and the Egger test for publication bias was nonsignificant (*p* = 0.54). By age category, studies of child obesity included two studies with an overall significant effect size of 0.90 (95% CI: 0.26, 1.54; *p* = 0.006; I^2^ = 22%), studies of adolescent obesity included six studies with an overall significant effect size of 1.57 (95% CI: 0.33, 2.81; *p* = 0.01; I^2^ = 96%), studies of adult obesity included 13 studies with an overall significant effect size of 1.63 (95% CI: 0.73, 2.52; *p* < 0.001; I^2^ = 97%), and studies of older adult obesity included eight studies with an overall nonsignificant effect size of 0.30 (95% CI: −0.03, 0.63; *p* = 0.08; I^2^ = 77%). The sensitivity analyses, after excluding one trial (Kim et al. [34]) with an extreme value that was notably an outlier, showed that the point estimates changed by −0.20 (SMD = 1.00; 95% CI: 0.58, 1.42).

**FIGURE 3:**
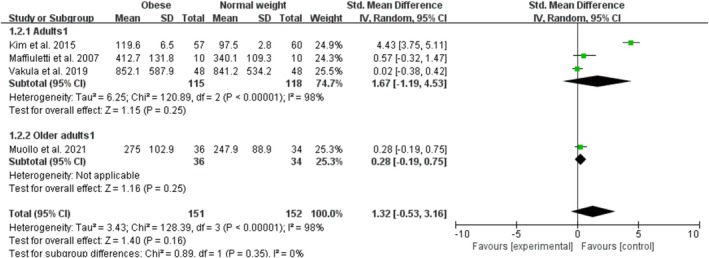
Forest plot of absolute measures of rapid knee extensor contractile function.

Obesity had a nonsignificant effect on absolute measures of rapid knee extensor contractile function with a combined effect of 1.32 (95% CI: −0.53, 3.16; *p* = 0.16; I^2^ = 98%) for the four studies included in the analysis (Figure 3). The funnel plot was asymmetrical, and the Egger test for publication bias was significant (*p* = 0.02). By age category, there were no studies on child or adolescent obesity and one study on older adult obesity. Adult obesity included three studies with an overall nonsignificant effect size of 1.67 (95% CI: −1.19, 4.53; *p* = 0.25; I^2^ = 98%). The sensitivity analyses, after excluding one trial (Kim et al. [34]) with an extreme value that was notably an outlier, showed that the point estimates changed by −1.15 (SMD = 0.17; 95% CI: −0.11, 0.46).

**FIGURE 4:**
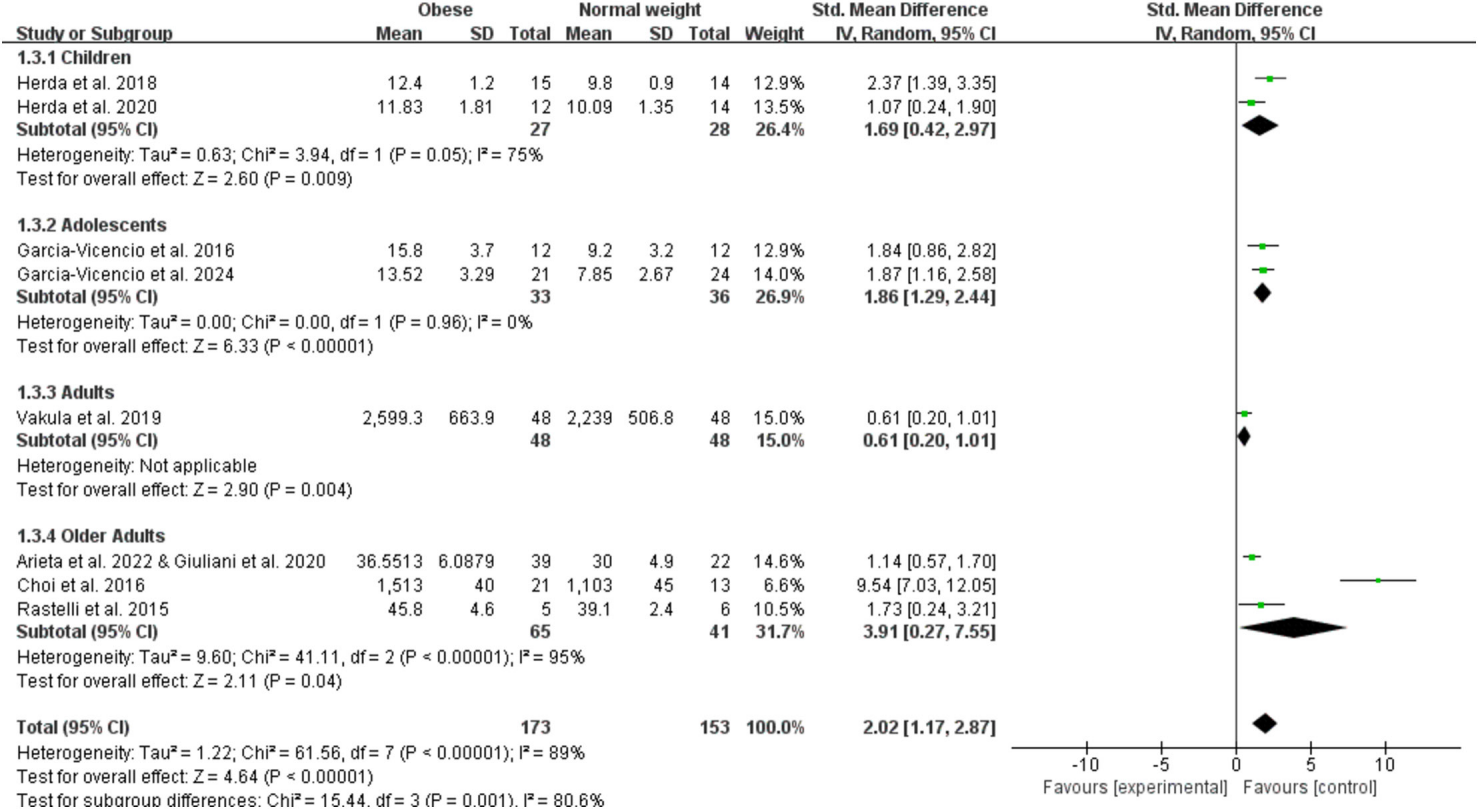
Forest plot of measures of knee extensor size.

Obesity significantly increased measures of knee extensor size with a combined effect of 2.02 (95% CI: 1.17, 2.87; *p* < 0.001; I^2^ = 89%) for the nine studies included in the analysis (Figure 4). The funnel plot was asymmetrical, and the Egger test for publication bias was significant (*p* < 0.001). By age category, there was one study on adult obesity, two studies on child obesity with an overall significant effect size of 1.69 (95% CI: 0.42, 2.97; *p* = 0.009; I^2^ = 75%), two studies on adolescent obesity with an overall significant effect size of 1.86 (95% CI: 1.29, 2.44; *p* < 0.001; I^2^ = 0%), and four studies on older adult obesity with an overall significant effect size of 3.91 (95% CI: 0.27, 7.55; *p* = 0.04; I^2^ = 95%). The sensitivity analyses, after excluding one trial (Choi et al. [15]), showed that the point estimates changed by −0.6 (SMD = 1.42; 95% CI: 0.91, 1.93).

### 3.6 Relative Measures of Knee Extensor Structure and Function

**FIGURE 5:**
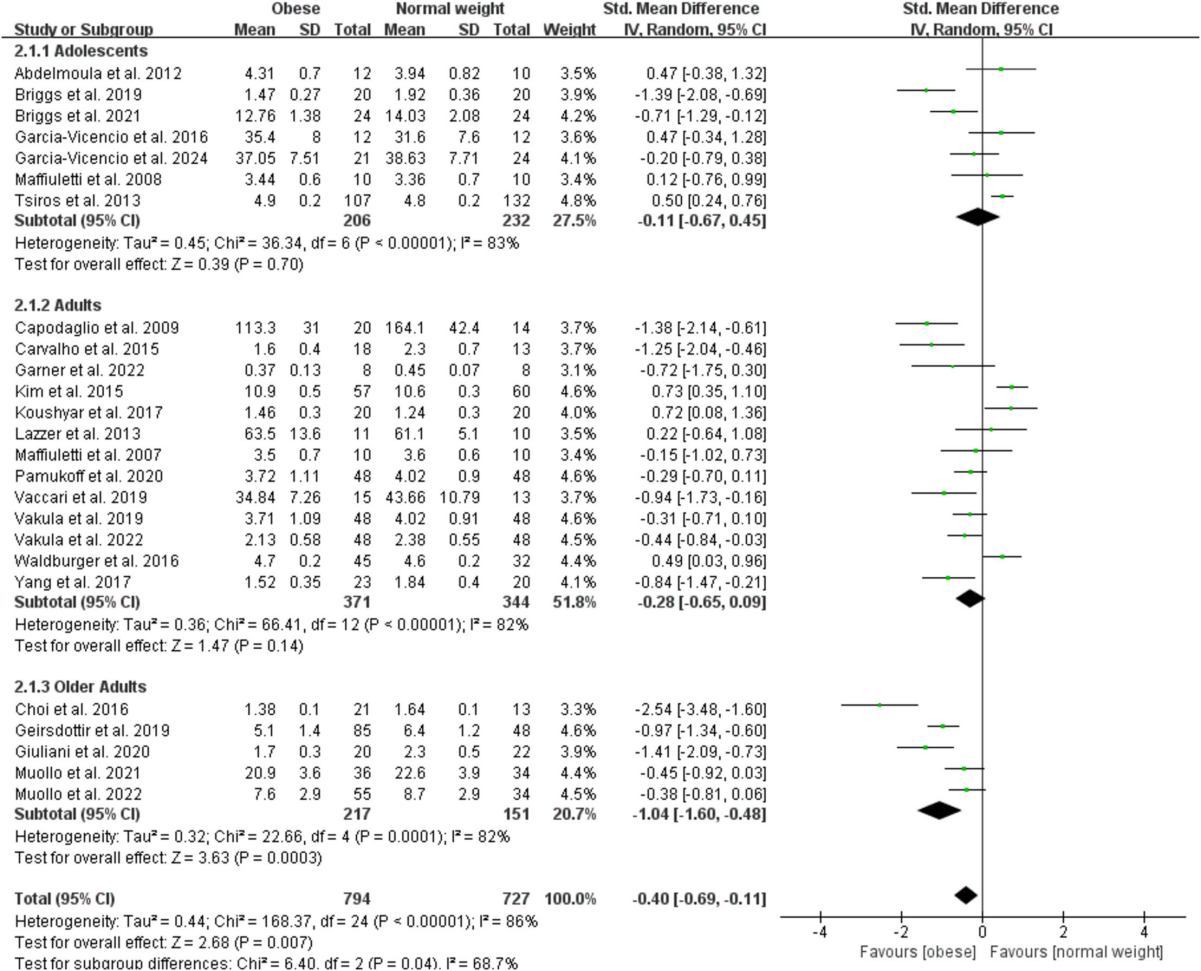
Forest plot of relative measures of maximal knee extensor contractile function.

Obesity significantly decreases relative measures of maximal knee extensor contractile function with a pooled effect of −0.40 (95% CI: −0.69, −0.11; *p* = 0.007; I^2^ = 86%) for the 25 studies (Figure 5). The funnel plot was symmetrical, and the Egger test for publication bias was nonsignificant (*p* = 0.15). By age category, there were no studies on child obesity, seven studies on adolescent obesity with an overall nonsignificant effect of −0.11 (95% CI: −0.67, 0.45; *p* = 0.70; I^2^ = 83%), 13 studies on adult obesity with a nonsignificant effect size of −0.28 (95% CI: −0.65, 0.09; *p* = 0.14; I^2^ = 82%), and five studies on older adult obesity with a significant effect of −1.04 (95% CI: −1.60, −0.48; *p* < 0.001; I^2^ = 86%) on relative measure of maximal knee extensor function. The sensitivity analyses, after excluding one trial (Choi et al. [15]), showed that the point estimates changed by 0.08 (SMD = −0.32; 95% CI: −0.60, −0.05).

**FIGURE 6:**
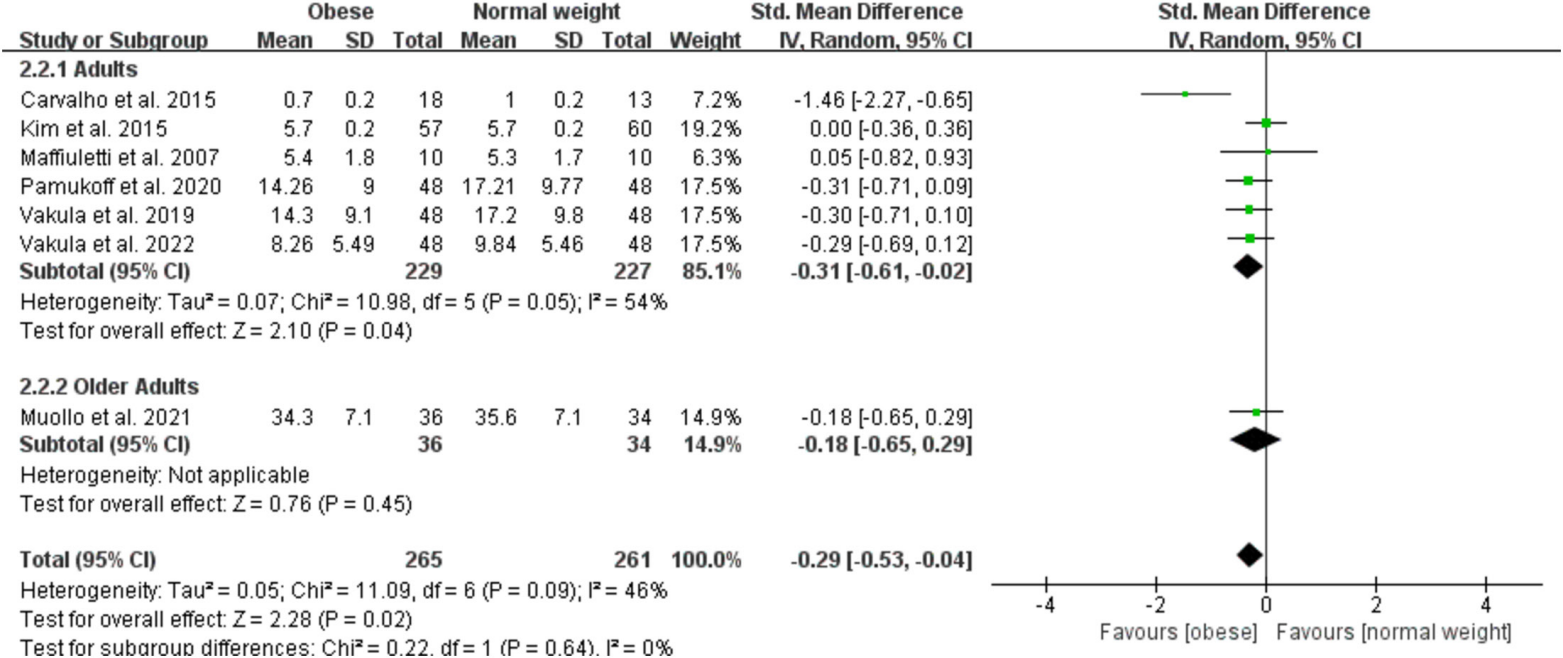
Forest plot of relative measures of rapid knee extensor contractile function.

Obesity significantly decreases relative measures of rapid knee extensor contractile function with a pooled effect of −0.29 (95% CI: −0.53, −0.04; *p* = 0.02; I^2^ = 46%) for seven studies (Figure 6). The funnel plot was symmetrical, and the Egger test for publication bias was nonsignificant (*p* = 0.15). By age category, there were no studies on child or adolescent obesity, four studies on adult obesity with an overall significant effect of −0.31 (95% CI: −0.61, −0.02; *p* = 0.04; I^2^ = 54%), and one study on the effect of older adult obesity on relative measures of rapid knee extensor function. The sensitivity analyses, after excluding one trial (Carvalho et al. [41]), showed that the point estimates changed by 0.09 (SMD = −0.20; 95% CI: −0.37, −0.02).

**FIGURE 7:**
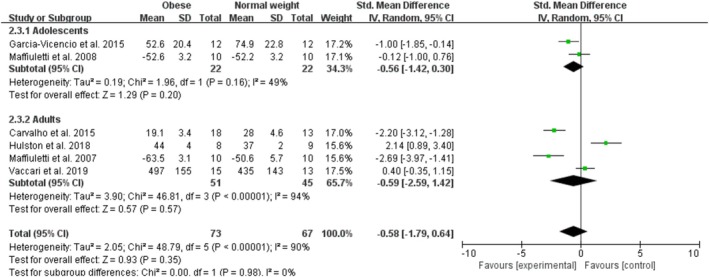
Forest plot of relative measures of knee extensor fatigue.

Obesity had no effect on relative measures of knee extensor fatigue with a pooled effect of −0.58 (95% CI: −1.79, 0.64; *p* = 0.35; I^2^ = 90%) for six studies (Figure 7). The funnel plot was symmetrical, and the Egger test for publication bias was nonsignificant (*p* = 0.14). By age category, there were no studies on child obesity, two studies on adolescent obesity with an overall nonsignificant effect of −0.56 (95% CI: −1.42, 0.30; *p* = 0.20; I^2^ = 49%), four studies on adult obesity with an overall nonsignificant effect of −0.59 (95% CI: −2.59, 1.42; *p* = 0.57; I^2^ = 94%), and no studies on older adult obesity on relative measures of knee extensor fatigue. The sensitivity analyses, after excluding one trial (Maffiuletti et al. [9]), showed that the point estimates changed by 0.39 (SMD = −0.19; 95% CI: −1.43, 1.04).

**FIGURE 8:**
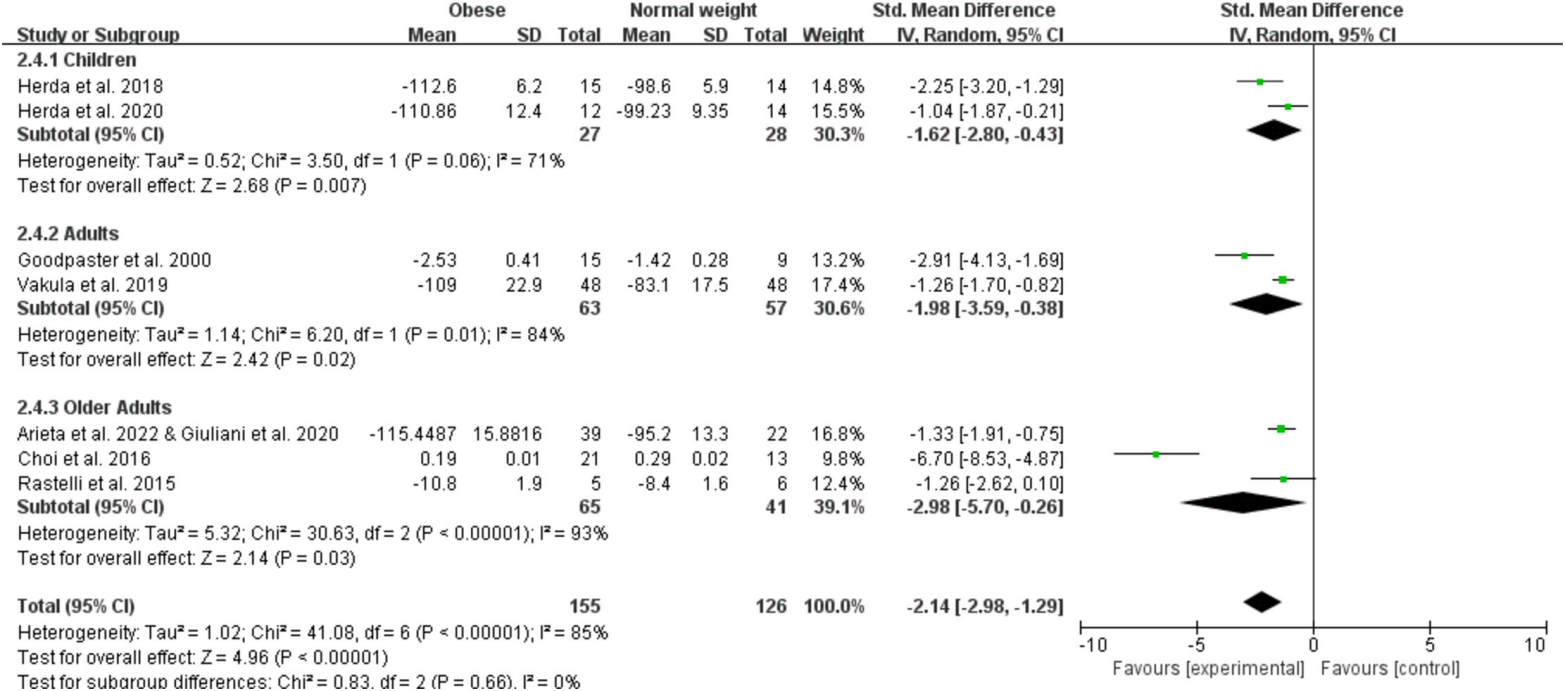
Forest plot of measures of knee extensor muscle quality.

Obesity significantly decreases measures of knee extensor muscle quality with a pooled effect of −2.14 (95% CI: −2.98, −1.29; *p* < 0.001; I^2^ = 85%) for eight studies (Figure 8). The funnel plot was asymmetrical, and the Egger test for publication bias was significant (*p* = 0.002). By age category, there were two studies on child obesity with an overall significant effect of −1.62 (95% CI: −2.80, −0.43; *p* = 0.007; I^2^ = 71%), no studies on adolescent obesity, two studies on adult obesity with an overall significant effect of −1.98 (95% CI: −3.59, −0.38; *p* = 0.02; I^2^ = 84%), and five studies on older adult obesity with an overall significant effect of −2.98 (95% CI: −5.70, −0.26; *p* = 0.03; I^2^ = 93%) on measures of knee extensor muscle quality. The sensitivity analyses, after excluding one trial (Choi et al. [15]), showed that the point estimates changed by 0.59 (SMD = −1.55; 95% CI: −2.01, −1.09).

## 4 Discussion

The aim of this systematic review and meta‐analysis was to evaluate the association of obesity with absolute and relative measures of knee extensor muscle structure (muscle size and quality) and contractile function (maximal force/torque, rapid torque, and fatigue) in children, adolescents, adults, and older adults. Overall, we included 37 studies in the systematic review and 31 studies into the meta‐analysis. Our results, as a whole, suggest that knee extensor structure and function are altered with obesity. In particular, obesity significantly increased absolute maximal measures of knee extensor contractile force/torque and muscle size. Conversely, obesity decreased relative maximal measures of knee extensor contractile force/torque and muscle quality. Importantly, the association of obesity with knee extensor structure and function differed by age category.

To the best of our knowledge, this is the first study to systematically review and analyze the association of obesity with knee extensor muscle structure and function in children, adolescents, adults, and older adults. Overall, obesity increased absolute maximal measures of knee extensor contractile function, suggesting the peak torque or force production capabilities of the knee extensors, without respect to body size, are increased with obesity. This increase in absolute maximal contractile function is likely due to the increase in body mass that accompanies obesity, which, while weight bearing, acts as an overload stimulus for antigravity musculature such as the knee extensors.

While this increase in contractile function has been suggested as a favorable adaptation with obesity [7], our results indicate that the effect of obesity on absolute maximal knee extensor contractile function varies significantly with age. In adolescents and adults, obesity was associated with significant increases in absolute contractile function, likely due to the mechanical overload provided by excess body mass. However, in older adults, this effect was nonsignificant, consistent with findings from previous reviews [17]. This diminished response in older adults may be attributed to age‐related factors such as reduced physical activity, sarcopenia, and chronic inflammation, which impair the ability to maintain or increase muscle mass and function despite the mechanical stimulus of excess weight. [42, 43] Notably, while obesity significantly decreased measures of muscle quality in children and adults, the effect was nonsignificant in older adults. This pattern suggests that younger individuals with obesity experience more pronounced impairments in muscle composition, such as increased intramuscular fat infiltration, whereas the impact may be masked or overridden by age‐related declines in muscle mass and function in older adults. These findings emphasize the importance of considering age‐related differences when interpreting the impact of obesity on muscle performance and highlight the need for interventions targeting age‐specific deficits in muscle structure and function.

Although absolute measures of knee extensor contractile function tend to increase with obesity, relative measures may be more critical to activities of daily living (ADLs), mobility, and fall recovery. Overall, obesity decreased relative measures of maximal knee extensor function, suggesting that when scaled allometrically, the torque producing capabilities of the knee extensors is reduced. This reduction in knee extensor contractile function is important considering their contribution to mobility and risk for knee osteoarthritis [44, 45, 46]. Furthermore, our subgroup analysis suggested that the effect of obesity on relative knee extensor function was greater in older adults. A result, which may be influenced by a decline in central neurological dysfunction as well as sarcopenia [47]. While further studies are needed to pinpoint the mechanisms behind this altered contractile function, surely, the increased muscle size but decreased muscle quality we found plays a role.

While absolute measures of knee extensor contractile function are often increased with obesity, relative measures provide a more meaningful assessment of functional capacity, as they account for the disproportionate impact of excess body mass on physical performance. This study observed significant reductions in relative knee extensor strength, power, and muscle quality in individuals with obesity, deficits that are directly relevant to functional limitations in ADLs. These findings are in alignment with the broader body of literature examining the interplay between obesity, muscle function, and physical performance. Tsiros et al. [48] highlighted that obesity in children is not merely a risk factor for future health complications but also a current disability, manifesting as profound limitations in physical function and independence during ADLs. Importantly, this emphasizes that reductions in relative muscle strength and quality, as observed in this study, have real‐world consequences for mobility and participation in daily tasks even at a young age. Furthermore, these functional impairments can compound over time, reinforcing a cycle of reduced physical activity and increasing musculoskeletal deterioration [48]. Similarly, Tareque et al. [49] demonstrated that obesity in older adults is associated with significantly longer periods of functional impairment and dependency in ADLs compared to their normal‐weight counterparts. These findings complement the current study's results by illustrating how reductions in relative muscle strength and quality not only predict but actively contribute to the progressive loss of independence and heightened fall risk in obese older adults. The inclusion of relative measures in such analyses provides critical insight into the mechanisms underlying these functional declines, which are often masked when assessing absolute measures alone [49]. Collectively, these results emphasize that deficits in relative muscle function and quality, as observed in this study, represent a critical link connecting obesity to physical limitations across the lifespan. By addressing these impairments, particularly through interventions targeting improvements in relative muscle efficiency, there is potential to mitigate the long‐term impact of obesity on mobility and independence, ultimately improving quality of life for obese individuals.

In addition to the observed deficits in relative muscle function, the impact of obesity on muscle fiber phenotype warrants further consideration. Obesity has been shown to alter the distribution of muscle fiber types, increasing the proportion of fast‐twitch Type IIx fibers while reducing the prevalence of slow‐twitch Type I fibers [50, 51]. This shift compromises oxidative capacity and fatigue resistance, making muscles less capable of sustaining prolonged activity or repetitive contractions. For example, muscles with a higher proportion of Type IIx fibers are more susceptible to fatigue, particularly during concentric contractions critical for daily mobility tasks [52]. Further, the accumulation of intramuscular fat, commonly observed in obese individuals, disrupts muscle contractile properties by increasing stiffness and reducing force output at the cellular level [53]. These changes at the muscle fiber level may contribute to the greater fatigue rates and diminished functional capacity observed in this study, especially in the context of activities requiring sustained or repetitive effort. Considered together, these findings highlight the importance of considering muscle fiber‐level adaptations when interpreting the association of obesity with muscle performance. Interventions aimed at improving muscle quality, such as resistance training that promotes Type I fiber development and reduces intramuscular fat infiltration, could offer promising strategies to enhance mobility and physical independence for individuals with obesity.

As a whole, obesity increased the size of the knee extensors, but decreased muscle quality. Our finding suggests that individuals with obesity have larger but lower quality knee extensor muscles when compared to individuals without obesity. The increase in the size of load‐bearing musculature such as the knee extensors is accepted in the literature as an adaptation to the increased mass from excessive adipose tissue with obesity [17]. However, it should be noted that the percentage of body mass coming from body fat was approximately 40% in individuals with obesity and 24% in individuals with normal weight on average, and therefore, the relative amount of fat‐free mass in the body is reduced with obesity. While the percentage differences in fat‐free mass may in part explain the decrease in relative contractile function we observe in individuals with obesity, our results would also indicate that reduced muscle quality in this population should also be considered when explaining the decrements in relative contractile function.

The majority of studies reporting the effect of obesity on muscle quality either directly or indirectly measured the fat composition of the knee extensor muscles. In half of the studies included in our meta‐analysis [11, 40, 54], muscle quality was measured via ultrasound echo intensity, an alternate measure of intermuscular fat accumulation [15]. Therefore, our results suggest that the relative contribution of contractile tissue in the knee extensors is reduced with obesity. This increase in noncontractile adipose tissue within the muscle fiber is negatively associated with contractile function [15, 53] and knee extensor function while walking [11]. However, the inclusion of fat into the muscle may explain, in part, the increase in muscle size and decrease in relative force seen with obesity.

Interestingly, we found no significant effect of obesity on absolute measures of rapid knee extensor contractile function. Although measures of rapid knee extensor contractile function may be more relevant assessments to ADLs such as walking, they are underreported in the literature. The limited number of studies included in our analysis appear to suggest that absolute rapid contractile function is increased with obesity, whereas relative contractile function of the knee extensors is decreased with obesity. However, additional research is needed to reach such conclusions. It may be argued that the nonsignificant effect of obesity on rapid knee extensor function we reported was influenced by our inclusion of both isometric RTD and isokinetic power in our meta‐analysis of rapid contractile function. Although RTD and power are both rate‐based assessments of contractile function, they utilize different muscle actions (i.e., isometric vs. concentric), and differences in muscle contractile function may have been concealed by our methods. Future research with increased consistency in the outcomes reported is needed to better understand the effect of obesity on rapid knee extensor contractile function.

Although fatigue of the knee extensors has been suggested as a limitation during ADLs, we found that obesity had no overall effect on measures of knee extensor fatigue. Of the six studies we included in our analysis, four reported muscle fatigue was decreased with obesity (i.e., reduced ability), suggesting the association of obesity with knee extensor fatigue is undecided. Among individuals with obesity, peripheral mechanisms such as the greater absolute maximal voluntary torque and proportion of fast‐twitch muscle fibers in the knee extensors are suggested as factors that may contribute to increased fatigue with obesity [25, 52, 55]. However, additional research is needed to reinforce these findings. Alternatively, our nonsignificant result could have been influenced by factors such as age, sex, physical activity level, contraction type, testing methods, and severity of obesity, among other factors, which were not accounted for in our analysis. Future studies with improved controls for such factors are needed to fully understand the effect of obesity on muscle fatigue.

The following limitations should be considered when interpreting the results of this study. Firstly, the studies that were included in this systematic review and meta‐analysis were limited to those published in peer‐reviewed journals in the English language. Although excluding unpublished data may increase publication bias, we searched a large number of databases and excluded duplicate data in order to limit the bias introduced into the review [56]. Secondly, in a limited number of studies, data were combined by group (e.g., male and female), testing speed, and/or testing angle. This allowed us to include all eligible participants and experimental trials without introducing sampling bias; however, it may have disguised differences in knee extensor function and structure that may have existed. Thirdly, our review and analysis of knee extensor function was limited to concentric and isometric muscle actions. Yet, while walking, the knee extensor predominantly uses an eccentric muscle action to modulate forces acting on the knee joint and support the body [57, 58]. We found no studies that reported on eccentric muscle function, and thus, future studies are needed to examine the effect of obesity on eccentric muscle contractile function, as it may be more applicable to functional movements such as walking. Also, the analysis exhibited high heterogeneity (I^2^ > 90%) across studies. Despite employing subgroup analyses by age, a random‐effects model, and robustness checks (e.g., sensitivity analysis and outlier removal), heterogeneity remained substantial. This likely reflects methodological variability among the included studies, such as differences in muscle contraction types (isometric, isotonic, and isokinetic) and measurement devices used to assess knee extensor function. Although subgroup analyses based on these methodological differences could potentially reduce heterogeneity, the limited number of studies available precluded such an analysis in this study. Future meta‐analyses would benefit from larger datasets that allow for stratification based on measurement methods and protocols, enabling more accurate and reliable interpretations of pooled results.

Additionally, our decision to exclude participants categorized as overweight, focusing solely on comparisons between OB and NW groups, may limit the generalizability of our findings. From a clinical standpoint, understanding how overweight status affects muscle structure and function could provide valuable insights into early intervention opportunities before the progression to obesity. Future research should aim to include overweight populations and report data separately rather than aggregating overweight and obese groups, as distinct physiological differences may exist between them. Moreover, we used BMI as the sole criterion for classifying obesity. While BMI offers standardized comparability across studies, it does not differentiate between lean mass and fat mass. This limitation may have influenced participant classification accuracy, particularly in individuals with high muscle mass and low fat mass that may not be reflected accurately by BMI. The inclusion of %BF in future studies may improve the specificity of obesity classification and help clarify the relationship between obesity and knee extensor muscle function. Nonetheless, the results of this systematic review and meta‐analysis will provide a crucial foundation for advancing standardized methodologies and enhancing the reliability of future research.

The findings of this systematic review and meta‐analysis provide critical clinical implications for the management and treatment of individuals with obesity. While obesity increases the absolute size and maximal contractile capacity of knee extensors, the concurrent reductions in relative contractile function and muscle quality underscore the heightened vulnerability to mobility impairments and functional limitations. These deficits are particularly relevant to ADLs and postural stability, where efficient relative muscle function is paramount. Moreover, the pronounced effect of obesity on relative function in older adults highlights the compounded impact of potential sarcopenia, reduced physical activity, and intramuscular fat infiltration with advancing age. Interventions aimed at improving relative muscle function and quality, such as targeted resistance training, dietary modifications to reduce adiposity, and functional exercises tailored to daily movement patterns, may be essential to mitigate these deficits. Such strategies could enhance physical independence, reduce fall risk, and improve overall quality of life in individuals with obesity, especially in aging populations. This study provides a foundation for future research to explore specific interventions addressing these critical areas and to refine clinical approaches to obesity‐related muscle dysfunction.

## 5 Conclusion

In closing, the current systematic review and meta‐analysis provides evidence that obesity increases absolute maximal knee extensor function and size, but reduces relative maximal knee extensor function and quality. However, the effect of obesity on knee extensor size, quality, and absolute maximal contractile function appears to diminish with age. While the absolute torque producing capabilities and size of the knee extensors is increased with obesity, the relative reduction in knee extensor function and quality is of higher importance to individuals living with obesity while performing ADLs. Researchers and clinicians focusing on the treatment and management of obesity should consider interventions that improve the relative contractile function and muscle quality of the knee extensors for optimal practice. Finally, relative to the number of individuals affected by obesity, this systematic review and meta‐analysis was limited to a small number of studies. Additional research is needed to understand the effects of obesity on muscle structure and function.

## Conflicts of Interest

The authors declare no conflicts of interest.

## Supporting information


**Table S1** Modified epidemiological appraisal instrument.
**Table S2** List of all included studies (*n* = 37).
**Table S3** Participant characteristics and study details.
**Figure S1** Results of the methodological quality and risk of bias assessment.
