# Supplementary material for: Knee Extensor Structure and Function in Children, Adolescents, Adults, and Older Adults With Obesity: A Systematic Review and Meta‐Analysis
Source: Obes Rev. 2025 May 19;26(10):e13949. doi: 10.1111/obr.13949 (PMC12404886; doi:10.1111/obr.13949)
Supplement: Supplementary file 1 — Table S1 Modified epidemiological appraisal instrument. Table S2 List of all included studies (n = 37). Table S3 Participant characteristics and study details. Figure S1 Results of the methodological quality and risk of bias assessment. [file OBR-26-e13949-s001.pdf]

Knee Extensor Structure and Function in Children, Adolescents, Adults, and Older Adults with Obesity: A Systematic Review and Meta-analysis

Michael N. Vakula[1], Youngwook Kim[2], Eadric Bressel[1]

[1] Utah State University, Department of Kinesiology and Health Science, Logan, Utah, USA

[2] Soonchunhyang University, Department of Sports Medicine, Asan, South Korea

***Corresponding author: Youngwook Kim, PhD, ATC***

Department of Sports Medicine, Soonchunhyang University, 22, Soonchunhyang-ro, Asan-si, Chungcheongnam-do, Republic of Korea [31538]

Tel: +82 041-530-1338

Fax: +82 041-542-4615

E-mail: youngwookkim@sch.ac.kr

**Table S1** Modified epidemiological appraisal instrument

| | |
|---|---|
| **1. Study description** | |
| Hypothesis / aim / objectives | 1. Is the hypothesis/aim/objective of the study clearly described? |
| Outcome | 3. Are the main outcomes clearly described? |
| Study design | 4. Is the study design clearly described? |
| Study population | 5. Is the source of subject population (including sampling frame) clearly described?<br>6. Are the eligibility criteria for subject selection clearly described?<br>7. Are the participation rate(s) reported? Is ascertainment of record availability described?<br>8. Are the characteristics of study participants described? |
| Covariates and confounders | 11. Are the important covariates and confounders described in terms of individual variables?<br>12. Are the important covariates and confounders in terms of environment variables described? |
| Statistical Tests and Analysis Strategies | 13. Are the statistical methods clearly described? |
| Results | 14. Are the main findings of the study clearly described?<br>15. Does the study provide estimates of the random variability in the data for the main outcomes or exposures (i.e. confidence intervals, standard deviations)?<br>16. Does the study provide estimates of the statistical parameters (e.g. regression coefficients or parameter estimates such as odds ratio)?<br>17. Are sample size calculations performed and reported? |
| **2. Study's methodological quality** | |
| ***Subject selection*** | |
| Group comparability | 18. Is the comparison/reference group comparable to the exposed/intervention/case group? |
| Participation rate/record availability | 19. Is the participation rate adequate? Is the ascertainment of record availability adequate? |
| Time period | 20. Are the study subjects from different groups recruited over the same period? |
| Subject losses/unavailability of records | 21. Are subject losses or unavailable records after entry into the study taken into account? |
| ***Measurement quality*** | |
| Blind measurement | 29. Are the observers blinded to: subject groupings when the exposure/intervention assessment was made or the disease status of subjects when conducting exposure assessment? |
| Outcome | 31. Are the main outcome measures reliable?<br>32. Are the main outcome measures valid? |

| | |
|---|---|
| | 33. Are the methods of assessing the outcome variables standard across all groups? |
| ***Data analysis*** | |
| Observation period | 34. Are the observations taken over the same time for all groups? |
| Covariates and confounders | 35. Is prior history of disease and/or symptoms collected and included in the analysis? |
| | 36. Is there adequate adjustment for covariates and confounders in terms of individual variables in the analyses? |
| | 37. Is there adequate adjustment for covariates and confounders in terms of environment variables (other than exposure) in the analyses? |
| | 40. Are outcome data reported by levels of exposure? |
| | 41. Are the outcome/exposure data reported by subgroups of subjects? |
| ***Generalization of results*** | |
| | 42. Can the study results be applied to the eligible population? |
| | 43. Can the study results be applied to other relevant populations? |

The following items from the original EAI were removed from analysis, as they were not applicable to the included studies 2, 9, 10, 22, 23, 24, 25, 26, 27, 28, 30, 38 and 39. Each item was scored as "Yes" (score of 2), "Partial" (score of 1), "No" (score of 0), "Unable to determine" (score of 0), or "Not applicable" (item excluded).

**Table S2** List of all included studies (n = 37)


1. Abdelmoula A, Martin V, Bouchant A, et al. Knee extension strength in obese and nonobese male adolescents. *Appl Physiol Nutr Metab Physiol Appl Nutr Metab*. 2012;37(2):269-275. doi:10.1139/h2012-010

2. Arieta LR, Giuliani-Dewig HK, Gerstner GR, Mota JA, Ryan ED. Segmental bioelectrical impedance spectroscopy: A novel field assessment of muscle size and quality in normal weight and obese older men. *Exp Gerontol*. 2022;162:111745. doi:10.1016/j.exger.2022.111745

3. Briggs MS, Bout-Tabaku S, Buell J, White S, Rosenstein PF, Schmitt LC. A preliminary evaluation of the associations among functional performance tasks and quality of life in obese and healthy weight youth. *J Sports Sci*. 2019;37(1):20-28. doi:10.1080/02640414.2018.1479947

4. Briggs MS, Spech C, King R, et al. Obese Youth Demonstrate Altered Landing Knee Mechanics Unrelated to Lower-Extremity Peak Torque When Compared With Healthy Weight Youth. *J Appl Biomech*. 2021;37(2):109-117. doi:10.1123/jab.2020-0013

5. Capodaglio P, Vismara L, Menegoni F, Baccalaro G, Galli M, Grugni G. Strength characterization of knee flexor and extensor muscles in Prader-Willi and obese patients. *BMC Musculoskelet Disord*. 2009;10:47. doi:10.1186/1471-2474-10-47

6. Carvalho LP, Di Thommazo-Luporini L, Aubertin-Leheudre M, et al. Prediction of Cardiorespiratory Fitness by the Six-Minute Step Test and Its Association with Muscle Strength and Power in Sedentary Obese and Lean Young Women:  A Cross-Sectional Study. *PloS One*. 2015;10(12):e0145960. doi:10.1371/journal.pone.0145960

7. Choi SJ, Files DC, Zhang T, et al. Intramyocellular Lipid and Impaired Myofiber Contraction in Normal Weight and Obese Older Adults. *J Gerontol A Biol Sci Med Sci*. 2016;71(4):557-564. doi:10.1093/gerona/glv169

8. Duan X, Rhee J, Mehta RK, Srinivasan D. Neuromuscular Control and Performance Differences Associated With Gender and Obesity in Fatiguing Tasks Performed by Older Adults. *Front Physiol*. 2018;9:800. doi:10.3389/fphys.2018.00800

9. Garcia-Vicencio S, Martin V, Kluka V, et al. Obesity-related differences in neuromuscular fatigue in adolescent girls. *Eur J Appl Physiol*. 2015;115(11):2421-2432. doi:10.1007/s00421-015-3222-9

10. Garcia-Vicencio S, Coudeyre E, Kluka V, et al. The bigger, the stronger? Insights from muscle architecture and nervous characteristics in obese adolescent girls. *Int J Obes 2005*. 2016;40(2):245-251. doi:10.1038/ijo.2015.158

11. Garcia-Vicencio S, Martin V, Chalchat E, et al. Sex-Related Neuromuscular Adaptations to Youth Obesity: Force, Muscle Mass, and Neural Issues. *Adv Exp Med Biol*. 2024;1450:131-142. doi:10.1007/5584_2023_783

12. Garner RT, Weiss JA, Nie Y, et al. Effects of obesity and acute resistance exercise on skeletal muscle angiogenic communication pathways. *Exp Physiol*. 2022;107(8):906-918. doi:10.1113/EP090152

13. Geirsdottir OG, Chang M, Jonsson PV, Thorsdottir I, Ramel A. Obesity, Physical Function, and Training Success in Community-Dwelling Nonsarcopenic Old Adults. *J Aging Res*. Published online February 18, 2019:1-10. doi:10.1155/2019/5340328

14. Giuliani HK, Shea NW, Gerstner GR, Mota JA, Blackburn JT, Ryan ED. The Influence of Age and Obesity-Altered Muscle Tissue Composition on Muscular Dimensional Changes: Impact on Strength and Function. *J Gerontol A Biol Sci Med Sci*. 2020;75(12):2286-2294. doi:10.1093/gerona/glaa206

15. Goodpaster BH, Theriault R, Watkins SC, Kelley DE. Intramuscular lipid content is increased in obesity and decreased by weight loss. *Metabolism*. 2000;49(4):467-472. doi:10.1016/s0026-0495(00)80010-4

16. Hällsten K, Yki-Järvinen H, Peltoniemi P, et al. Insulin- and exercise-stimulated skeletal muscle blood flow and glucose uptake in obese men. *Obes Res*. 2003;11(2):257-265. doi:10.1038/oby.2003.39

17. Herda TJ, Ryan ED, Kohlmeier M, Trevino MA, Gerstner GR, Roelofs EJ. Examination of muscle morphology and neuromuscular function in normal weight and overfat children aged 7-10 years. *Scand J Med Sci Sports*. 2018;28(11):2310-2321. doi:10.1111/sms.13256

18. Herda TJ, Ryan ED, Kohlmeier M, et al. Muscle cross-sectional area and motor unit properties of the medial gastrocnemius and vastus lateralis in normal weight and overfat children. *Exp Physiol*. 2020;105(2):335-346. doi:10.1113/EP088181

19. Hulens M, Vansant G, Lysens R, Claessens AL, Muls E, Brumagne S. Study of differences in peripheral muscle strength of lean versus obese women: an allometric approach. *Int J Obes Relat Metab Disord J Int Assoc Study Obes*. 2001;25(5):676-681. doi:10.1038/sj.ijo.0801560

20. Hulston CJ, Woods RM, Dewhurst-Trigg R, et al. Resistance exercise stimulates mixed muscle protein synthesis in lean and obese young adults. *Physiol Rep*. 2018;6(14):e13799. doi:10.14814/phy2.13799

21. Kim B, Tsujimoto T, So R, et al. Weight loss may be a better approach for managing musculoskeletal conditions than increasing muscle mass and strength. *J Phys Ther Sci*. 2015;27(12):3787-3791. doi:10.1589/jpts.27.3787

22. Koushyar H, Nussbaum MA, Davy KP, Madigan ML. Relative Strength at the Hip, Knee, and Ankle Is Lower Among Younger and Older Females Who Are Obese. *J Geriatr Phys Ther 2001*. 2017;40(3):143-149. doi:10.1519/JPT.0000000000000086

23. Lazzer S, Salvadego D, Porcelli S, et al. Skeletal muscle oxygen uptake in obese patients: functional evaluation by knee-extension exercise. *Eur J Appl Physiol*. 2013;113(8):2125-2132. doi:10.1007/s00421-013-2647-2

24. Maffiuletti NA, Jubeau M, Munzinger U, et al. Differences in quadriceps muscle strength and fatigue between lean and obese subjects. *Eur J Appl Physiol*. 2007;101(1):51-59. doi:10.1007/s00421-007-0471-2

25. Maffiuletti NA, Jubeau M, Agosti F, De Col A, Sartorio A. Quadriceps muscle function characteristics in severely obese and nonobese adolescents. *Eur J Appl Physiol*. 2008;103(4):481-484. doi:10.1007/s00421-008-0737-3

26. Muollo V, Rossi AP, Zignoli A, et al. Full characterisation of knee extensors' function in ageing: effect of sex and obesity. *Int J Obes 2005*. 2021;45(4):895-905. doi:10.1038/s41366-021-00755-z

27. Muollo V, Zignoli A, Ghiotto L, et al. Knee flexor and extensor torque ratio in elderly men and women with and without obesity: a cross-sectional study. *Aging Clin Exp Res*. 2022;34(1):209-214. doi:10.1007/s40520-021-01884-1

28. Pamukoff DN, Vakula MN, Holmes SC, Shumski EJ, Garcia SA. Body mass index moderates the association between gait kinetics, body composition, and femoral knee cartilage characteristics. *J Orthop Res Off Publ Orthop Res Soc*. 2020;38(12):2685-2695. doi:10.1002/jor.24655

29. Rastelli F, Capodaglio P, Orgiu S, et al. Effects of muscle composition and architecture on specific strength in obese older women. *Exp Physiol*. 2015;100(10):1159-1167. doi:10.1113/EP085273

30. Rolland Y, Lauwers-Cances V, Pahor M, Fillaux J, Grandjean H, Vellas B. Muscle strength in obese elderly women: effect of recreational physical activity in a cross-sectional study. *Am J Clin Nutr*. 2004;79(4):552-557. doi:10.1093/ajcn/79.4.552

31. Tsiros MD, Coates AM, Howe PRC, et al. Knee extensor strength differences in obese and healthy-weight 10-to 13-year-olds. *Eur J Appl Physiol*. 2013;113(6):1415-1422. doi:10.1007/s00421-012-2561-z

32. Tsiros MD, Buckley JD, Olds T, et al. Impaired Physical Function Associated with Childhood Obesity: How Should We Intervene? *Child Obes Print*. 2016;12(2):126-134. doi:10.1089/chi.2015.0123

33. Vaccari F, Floreani M, Tringali G, De Micheli R, Sartorio A, Lazzer S. Metabolic and muscular factors limiting aerobic exercise in obese subjects. *Eur J Appl Physiol*. 2019;119(8):1779-1788. doi:10.1007/s00421-019-04167-w

34. Vakula MN, Fisher KL, Garcia SA, et al. Quadriceps Impairment Is Associated with Gait Mechanics in Young Adults with Obesity. *Med Sci Sports Exerc*. 2019;51(5):951-961. doi:10.1249/MSS.0000000000001891

35. Vakula MN, Garcia SA, Holmes SC, Pamukoff DN. Association between quadriceps function, joint kinetics, and spatiotemporal gait parameters in young adults with and without obesity. *Gait Posture*. 2022;92:421-427. doi:10.1016/j.gaitpost.2021.12.019

36. Waldburger R, Schultes B, Zazai R, et al. Comprehensive assessment of physical functioning in bariatric surgery candidates compared with subjects without obesity. *Surg Obes Relat Dis Off J Am Soc Bariatr Surg*. 2016;12(3):642-650. doi:10.1016/j.soard.2015.09.023

37. Yang F, Kim J, Yang F. Effects of obesity on dynamic stability control during recovery from a treadmill-induced slip among young adults. *J Biomech*. 2017;53:148-153. doi:10.1016/j.jbiomech.2017.01.021

**Table S3** Participant Characteristics and Study Details

| | Sample Size | | Age | | Sex | | BMI, kg/m$^2$ | | Body Fatt % | | Study Details | | | | |
|---|---|---|---|---|---|---|---|---|---|---|---|---|---|---|---|
| **Study** | **OB** | **NW** | **OB** | **NW** | **OB** | **NW** | **OB** | **NW** | **OB** | **NW** | **Limb analyzed** | **Testing device** | **Contraction Type** | **Joint Angle of Testing** | **Data Normalization** |
| Abdelmoula et al. 2012 | 12 | 10 | 14.2 ± 1.4 | 14.4 ± 0.7 | M 12 /F 0 | M 10 /F 0 | 34.1 ± 5.4 | 19.4 ± 1.7 | 40.6 ± 6.8 | 14.9 ± 3.7 | Dominant | Custom ergometer | Isometric | 60° | PT to FFM |
| Arieta et al. 2022 | 19 | 22 | 69.1 ± 2.5 | 69.4 ± 2.1 | M 19 /F 0 | M 22 /F 0 | 33.7 ± 3.7 | 23.3 ± 1.5 | 36.7 ± 3.7 | 24.4 ± 6.3 | Right | B-mode ultra sound | NA | NA | NA |
| Briggs et al. 2019 | 20 | 20 | 14.1 ± 2.1 | 14.1 ± 2.0 | M 13 /F 7 | M 13 /F 7 | 29.2 ± 2.2 | 20.4 ± 2.6 | 36.5 ± 7.3 | 21.9 ± 6.7 | Dominant | Dynamometer | Isokinetic (150°/s) | NA | PT to BM |
| Briggs et al. 2021 | 24 | 24 | 14.1 ± 2.0 | 14.2 ± 2.0 | M 16 /F 8 | M 16 /F 8 | 29.2 ± 2.0 | 20.1 ± 2.5 | 36.0 ± 7.0 | 21.0 ± 7.0 | Dominant | Dynamometer | Isokinetic (150°/s) | NA | PT to leg FFM |
| Capodaglio et al. 2009 | 20 | 14 | 29.1 ± 6.5 | 30.1 ± 4.7 | M 0 /F 20 | M 0 /F 14 | 38.1 ± 3.1 | 21.0 ± 1.6 | NA | NA | Right and Left | Dynamometer | Isokinetic (60, 180, 240°/s) | ROM 90° | PT to BM |
| Carvalho et al. 2015 | 18 | 13 | 35.0 ± 5.0 | 32.0 ± 5.0 | M 0 /F 18 | M 0 /F 13 | 42.2 ± 6.6 | 21.6 ± 2.0 | 47.9 ± 3.3 | 29.1 ± 3.3 | Dominant | Dynamometer | Isometric; Isokinetic (60°/s) | 90° (isometric); ROM 20 to 90° (isokinetic) | PT and PW to BM |

| | | | | | | | | | | | | | | | |
|---|---|---|---|---|---|---|---|---|---|---|---|---|---|---|---|
| Choi et al. 2016 | 21 | 13 | 69.0 ± 2.0 | 70.0 ± 2.0 | M 10 /F 11 | M 6 /F 7 | 30.0 ± 0.4 | 22.0 ± 0.52 | 39.0 ± 1.2 | 32.0 ± 1.7 | Right and Left | Dynamometer | Isokinetic | NA | PT to BW |
| Duan et al. 2018 | 29 | 30 | 72.4 ± 5.9 | 73.0 ± 5.5 | M 13 /F 16 | M 15 /F 15 | 37.0 ± 4.6 | 23.6 ± 1.8 | NA | NA | Dominant | Dynamometer | Isometric | 90° | NA |
| Garcia-Vicencio et al. 2015 | 12 | 12 | 13.9 ± 0.9 | 13.6 ± 0.8 | M 0 /F 12 | M 0 /F 12 | 32.1 ± 4.2 | 18.3 ± 1.3 | 38.2 ± 4.9 | 20.4 ± 2.9 | Right | Dynamometer | Isometric | 20, 40, 60, 70, 80, 90, 100° | NA |
| Garcia-Vicencio et al. 2016 | 12 | 12 | 13.9 ± 0.9 | 13.6 ± 0.8 | M 0 /F 12 | M 0 /F 12 | 32.1 ± 4.2 | 18.3 ± 1.3 | 38.2 ± 4.9 | 20.1 ± 2.9 | Right | B-mode ultra sound; Dynamometer | Isometric | 90° | PT to leg FFM |
| Garcia-Vicencio et al. 2024 | 21 | 24 | 13.9 ± 1.3 | 13.7 ± 1.0 | M 9 /F 12 | M 12 /F 12 | 32.8 ± 3.9 | 18.5 ± 1.5 | 38.7 ± 3.9 | 18.9 ± 5.1 | Right | B-mode ultra sound; Dynamometer | Isometric | 90° | PT to leg FFM |
| Garner et al. 2022 | 8 | 8 | 24.8 ± 4.5 | 21.5 ± 2.3 | M 4 /F 4 | M 4 /F 4 | 36.6 ± 5.0 | 21.7 ± 1.8 | NA | NA | Random | NA | Isotonic | NA | NA |
| Geirsdottir et al. 2019 | 85 | 48 | 73.3 ± 4.8 | 74.1 ± 5.8 | M 45 /F 40 | M 13 /F 35 | 33.9 ± NA | 23.0 ± 1.5 | 42.1 ± 5.8 | 33.5 ± 8.3 | NA | Dynamometer | Isometric | NA | PT to BW |
| Giuliani et al. 2020 | 20 | 22 | 69.0 ± 2.4 | 69.4 ± 2.1 | M 20 /F 0 | M 22 /F 0 | 34.0 ± 3.8 | 23.3 ± 1.5 | 37.0 ± 3.9 | 24.4 ± 6.3 | Right | B-mode ultra sound; Dynamometer | Isometric | 60° | PT to BM |

| | | | | | | | | | | | | | | | | |
|---|---|---|---|---|---|---|---|---|---|---|---|---|---|---|---|---|
| Goodpaster et al. 2000 | 15 | 9 | 36.0 ± 1.3 | 30.0 ± 2.0 | M 10 /F 5 | M 6 /F 3 | 33.6 ± 0.8 | 23.4 ± 1.1 | 35.3 ± 1.9 | 14.9 ± 1.8 | NA | Muscle biopsy | NA | NA | NA |
| Hällsten et al. 2003 | 9 | 11 | 30.0 ± 2.0 | 26.0 ± 1.0 | M 9 /F 0 | M 11 /F 0 | 36.0 ± 2.0 | 22.0 ± 1.0 | NA | NA | Right | Dynamometer | Isometric | 50° | NA |
| Herda et al. 2018 | 15 | 14 | 8.8 ± 0.4 | 8.7 ± 0.6 | M 5 /F 10 | M 6 /F 8 | 20.8 ± 1.2 | 15.8 ± 0.8 | 29.1 ± 2.9 | 18.0 ± 3.0 | Right knee | Dynamometer | Isometric | 90° | NA |
| Herda et al. 2020 | 12 | 14 | 8.8 ± 0.9 | 8.6 ± 1.1 | M 6 /F 6 | M 6 /F 8 | 21.0 ± 2.4 | 15.8 ± 1.4 | 29.3 ± 5.3 | 18.0 ± 5.2 | Right | Dynamometer | Isometric | 90° | NA |
| Hulens et al. 2001 | 173 | 80 | 39.9 ± 11.4 | 39.7 ± 12.2 | M 0 /F 173 | M 0 /F 80 | 37.8 ± 5.3 | 22.0 ± 2.2 | 46.7 ± 4.6 | 26.9 ± 5.0 | Right and Left | Dynamometer | Isokinetic | NA | NA |
| Hulston et al. 2018 | 8 | 9 | 24.0 ± 2.0 | 27.0 ± 1.0 | M 7 /F 1 | M 8 /F 1 | 33.6 ± 1.2 | 23.5 ± 0.6 | 29.5 ± 1.7 | 13.6 ± 1.9 | Dominant | Knee extension machine | Isotonic | NA | NA |
| Kim et al. 2015 | 57 | 60 | 47.1 ± 8.6 | 50.9 ± 9.3 | M 57 /F 0 | M 60 /F 0 | 33.3 ± 3.9 | 23.5 ± 1.3 | 26.6 ± 1.8 | 18.9 ± 0.5 | NA | Dynamometer | Isometric; Isokinetic (60°/s) | 60° (isometric) ; NA (isokinetic) | PT to leg FFM |
| Koushyar et al. 2017 | 20 | 20 | 45.3 ± 24.9 | 44.8 ± 25.8 | M 0 /F 20 | M 0 /F 20 | 32.8 ± 4.1 | 22.3 ± 4.6 | 39.0 ± 5.6 | 28.6 ± 7.6 | Right | Dynamometer | Isokinetic (75°/s) | NA | PT to BM |

| | | | | | | | | | | | | | | | | |
|---|---|---|---|---|---|---|---|---|---|---|---|---|---|---|---|---|
| Lazzer et al. 2013 | 11 | 10 | 29.5 ± 5.5 | 26.8 ± 10.2 | M 0 /F 11 | M 0 /F 10 | 43.2 ± 5.4 | 21.1 ± 2.4 | 53.2 ± 3.4 | 25.8 ± 4.9 | Right and Left | Force sensor and strap | Isometric | 70° | PT to leg FFM |
| Maffiuletti et al. 2007 | 10 | 10 | 25.3 ± 5.2 | 27.0 ± 4.1 | M 10 /F 0 | M 10 /F 0 | 41.3 ± 5.4 | 22.6 ± 1.1 | NA | NA | Right | Dynamometer | Isometric; Isokinetic (60, 120, 180°/s) | 40, 60, 80° (isometric); ROM 0 to 90° (isokinetic) | PT to FFM |
| Maffiuletti et al. 2008 | 10 | 10 | 15.6 ± 1.2 | 14.9 ± 1.1 | M 10 /F 0 | M 10 /F 0 | 34.0 ± 3.0 | 19.0 ± 1.0 | NA | NA | Right | Dynamometer | Isometric; Isokinetic (180°/s) | 40 and 80° (isometric); ROM 0 to 90° (isokinetic) | PT to FFM |
| Muollo et al. 2021 | 36 | 34 | 68.2 ± 5.3 | 69.4 ± 5.4 | M 18 /F 18 | M 18 /F 16 | 33.8 ± 3.6 | 23.6 ± 1.7 | 44.4 ± 8.1 | 29.4 ± 5.1 | Right | Dynamometer | Isometric; Isokinetic (60, 90, 150, 210°/s) | 30, 60, 75, 90° | PT to leg FFM |
| Muollo et al. 2022 | 55 | 34 | 67.8 ± 5.3 | 69.4 ± 5.4 | M 25 /F 30 | M 18 /F 16 | 33.0 ± 3.5 | 23.6 ± 1.7 | NA | NA | Right | Dynamometer | Isokinetic (60, 90, 150, 180, 210°/s) | NA | PT to leg FFM |
| Pamukoff et al. 2020 | 48 | 48 | 22.8 ± 3.5 | 22.0 ± 2.8 | M 24 /F 24 | M 24 /F 24 | 33.1 ± 4.1 | 21.6 ± 1.8 | 37.9 ± 7.1 | 19.9 ± 6.5 | Dominant | Dynamometer | Isometric | 60° | PT and RTD to FFM |
| Rastelli et al. 2015 | 5 | 6 | 72.4 ± 2.3 | 72.7 ± 1.9 | M 0 /F 5 | M 0 /F 6 | 36.8 ± 1.9 | 24.3 ± 1.8 | 41.9 ± 2.9 | 33.4 ± 1.9 | Dominant | Dynamometer | Isometric (90°); Isokinetic (60, 120, 180, 240°/s) | 90° | NA |

| | | | | | | | | | | | | | | | |
|---|---|---|---|---|---|---|---|---|---|---|---|---|---|---|---|
| Rolland et al. 2004 | 215 | 598 | 80.0 ± 3.5 | 80.7 ± 4.1 | M 0 /F 215 | M 0 /F 598 | 31.9 ± 2.6 | 21.6 ± 1.8 | 45.2 ± 4.4 | 30.2 ± 7.5 | Right and Left | Electronic statergometer | Isokinetic | UTD | NA |
| Tsiros et al. 2013 | 107 | 132 | 11.8 ± 0.1 | 12.0 ± 0.1 | M 56 / F 51 | M 76 /F 56 | 29.6 ± 0.4 | 18.2 ± 0.2 | 45.4 ± 0.5 | 21.7 ± 0.6 | Right and Left | Dynamometer | Isometric; Isokinetic (60°/s) | 90° (isometric); ROM 95° (isokinetic) | PT to FFM |
| Tsiros et al. 2016 | 107 | 132 | 11.8 ± 0.1 | 12.0 ± 0.1 | M 56 / F 51 | M 76 /F 56 | 29.6 ± 0.4 | 18.2 ± 0.2 | 45.4 ± 0.5 | 21.7 ± 0.6 | Right and Left | Dynamometer | Isokinetic (60°/s) | ROM 95° | PT to FFM |
| Vaccari et al. 2019 | 15 | 13 | 25.2 ± 6.8 | 26.5 ± 8.2 | M 12 /F 3 | M 10 /F 3 | 43.1 ± 7.5 | 21.9 ± 3.2 | 44.7 ± 6.9 | 20.1 ± 5.2 | Right | Custom ergometer | Isometric | 70° | PT to thigh muscle volume |
| Vakula et al. 2019 | 48 | 48 | 22.8 ± 3.5 | 22.0 ± 2.6 | M 24 /F 24 | M 24 /F 24 | 33.1 ± 4.1 | 21.6 ± 1.7 | 37.9 ± 7.2 | 19.9 ± 6.6 | Dominant | Dynamometer | Isometric | 60° | PT and RTD to FFM |
| Vakula et al. 2022 | 48 | 48 | 21.9 ± 3.2 | 22.9 ± 3.1 | M 24 /F 24 | M 24 /F 24 | 33.0 ± 3.2 | 21.6 ± 3.2 | 37.9 ± 7.0 | 19.9 ± 7.0 | Dominant | Dynamometer | Isometric | 60° | PT and RTD to FFM x height |
| Waldburger et al. 2016 | 45 | 32 | 35.0 ± 1.7 | 38.7 ± 2.1 | M 12 /F 33 | M 7 /F 25 | 42.6 ± 0.9 | 23.0 ± 0.4 | 45.7 ± 0.9 | 25.2 ± 1.0 | Right and Left | Dynamometer | Isometric | 90° | PT to FFM |
| Yang et al. 2017 | 23 | 20 | 24.9 ± 5.7 | 23.6 ± 4.1 | M 15 /F 8 | M 6 /F 14 | 35.1 ± 17.6 | 21.6 ± 2.4 | 37.7 ± 6.1 | 20.3 ± 5.8 | Right | Dynamometer | Isometric | NA | PT to BM |

*OB obese*, *NW normal weight*, *PT* peak torque, *PW* power, *RTD* rate of torque development, *FFM* fat free mass, *BF* body fat, *BM* body mass, *NA* not availiable.

# Fig. S1 Results of the methodological quality and risk of bias assessment

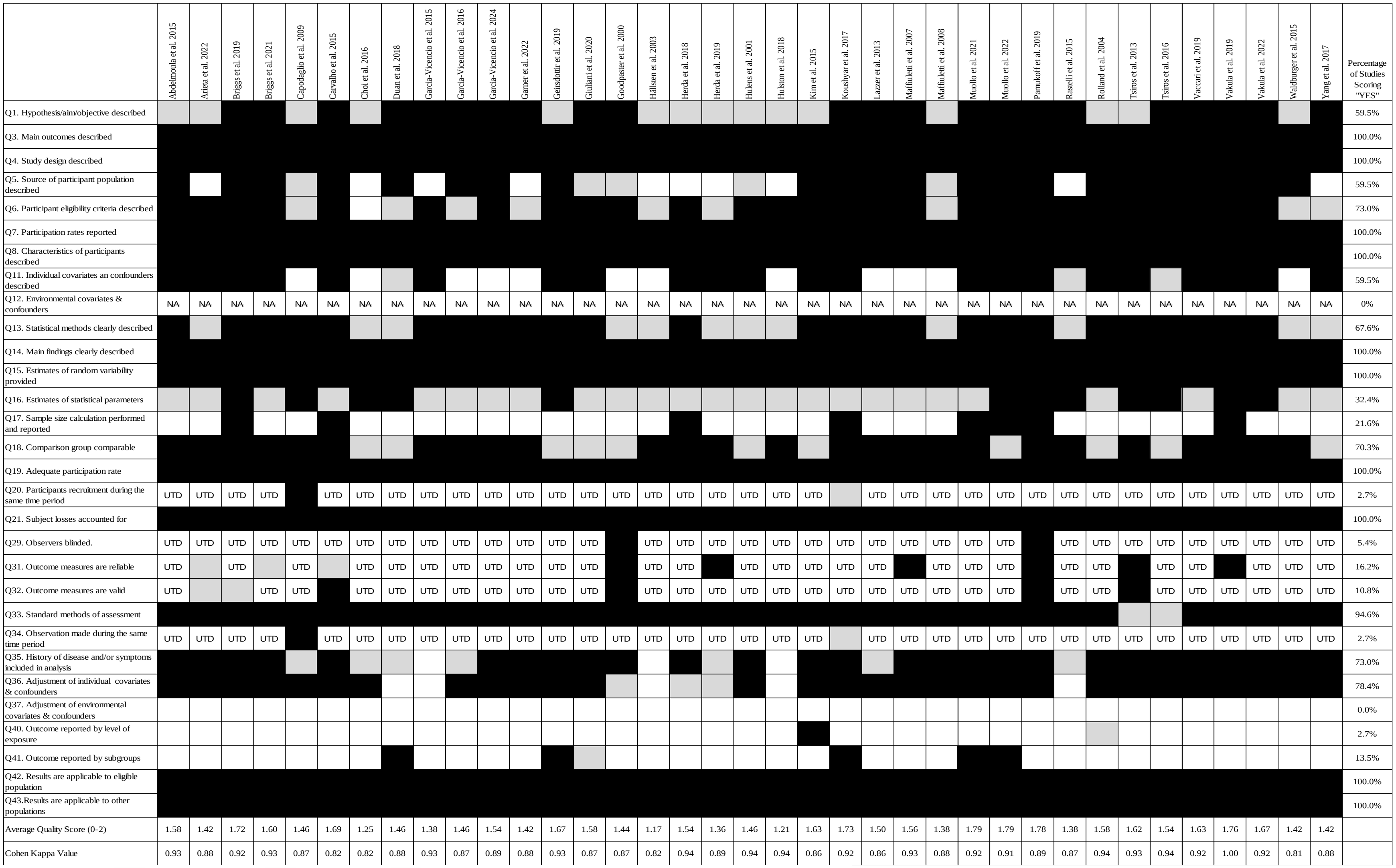


"Yes" (2) = ■; "Partial" (1) = ▒; "No" (0) = □ ; "Unable to determine"= UTD; "Not applicable" = ~~NA~~
